# Utilization of a mental health collaborative care model among patients who require interpreter services

**DOI:** 10.1186/s13033-016-0044-z

**Published:** 2016-02-29

**Authors:** Jane W. Njeru, Ramona S. DeJesus, Jennifer St. Sauver, Lila J. Rutten, Debra J. Jacobson, Patrick Wilson, Mark L. Wieland

**Affiliations:** Division of Primary Care Internal Medicine, Department of Medicine, Mayo Clinic, 200 First Street SW, Rochester, MN 55905 USA; Robert D and Patricia E Kern Center of the Science of Health Care Delivery, Mayo Clinic, Rochester, MN USA; Department of Health Sciences Research, Mayo Clinic, Rochester, MN USA; Division of Biomedical Statistics and Informatics, Department of Health Sciences Research, Mayo Clinic, Rochester, MN USA

**Keywords:** Collaborative care management, Depression, Interpreter services, Limited english proficiency, Mental health

## Abstract

**Background:**

Immigrants and refugees to the United States have a higher prevalence of depression compared to the general population and are less likely to receive adequate mental health services and treatment. Those with limited English proficiency (LEP) are at an even higher risk of inadequate mental health care. Collaborative care management (CCM) models for depression are effective in achieving treatment goals among a wide range of patient populations, including patients with LEP. The purpose of this study was to assess the utilization of a statewide initiative that uses CCM for depression management, among patients with LEP in a large primary care practice.

**Methods:**

This was a retrospective cohort study of patients with depression in a large primary care practice in Minnesota. Patients who met criteria for enrollment into the CCM [with a provider-generated diagnosis of depression or dysthymia in the electronic medical records, and a Patient Health Questionnaire-9 (PHQ-9) score ≥10]. Patient-identified need for interpreter services was used as a proxy for LEP. Rates of enrollment into the DIAMOND (Depression Improvement Across Minnesota, Offering A New Direction) program, a statewide initiative that uses CCM for depression management were measured. These rates were compared between eligible patients who require interpreter services versus patients who do not.

**Results:**

Of the 7561 patients who met criteria for enrollment into the DIAMOND program during the study interval, 3511 were enrolled. Only 18.2 % of the eligible patients with LEP were enrolled into DIAMOND compared with the 47.2 % of the eligible English proficient patients. This finding persisted after adjustment for differences in age, gender and depression severity scores (adjusted OR [95 % confidence interval] = 0.43 [0.23, 0.81]).

**Conclusions:**

Within primary care practices, tailored interventions are needed, including those that address cultural competence and language navigation, to improve the utilization of this effective model among patients with LEP.

## Background

Immigrants and refugees in developed countries, including the United States (US), have a higher prevalence of depression compared to the general population [1–5]. Several explanations have been proposed to account for this observation [1, 6]. Yet, they are less likely to receive appropriate mental health services and treatment, and when received, these services often do not meet the minimum accepted standards of care in the US [7–9]. Disparities in depression treatment among racial and ethnic minority groups, including immigrant and refugees as a whole, are well documented [10, 11]. However, less studied is the subset of this population who have limited English proficiency (LEP); a demographic whose population in the US increased by 80 % in the past two decades [12]. LEP persons include anyone above the age of five who reported speaking English less than “very well”, as classified by the US Census Bureau [13]. LEP has been associated with health disparities for the treatment of many chronic diseases [14, 15], and while the use of interpreters for health care interactions mitigates some of these disparities, it does not entirely eliminate them [16]. Among racial and ethnic minority groups, patients with LEP are at an even higher risk of inadequate mental health services [17].

Integrated behavioral health and care coordination systems are increasingly being adapted within primary care practices for patients with complex medical and psychosocial needs [18–20]. The collaborative care management (CCM) model for depression is one such multicomponent, health care system-level intervention that uses case managers to link patients seen by their primary care providers with mental health specialists [21]. This approach has led to higher utilization rates of anti-depressant medications and disease remission, compared to usual care, among both the general population and ethnic/racial minority groups [22–25]. It potentially may even ameliorate disparities in depression management among this group [26].

A recent systematic review suggested that LEP is associated with underutilization of mental health services [27]. It is therefore conceivable that the more proactive referral and engagement processes inherent to mental health CCMs, may improve utilization. The effectiveness of these models has also been demonstrated among patients with LEP in the US [28]. However, we found no published reports describing the degree of participation among patients with LEP in mental health CCM. To ensure that patients with LEP benefit fully from mental health CCMs, understanding their utilization patterns of this model is an important first step towards reducing disparities in mental health outcomes [29]. The purpose of this study was to assess the utilization of a CCM for depression among patients with LEP in a large primary care practice.

## Methods

### Setting and mental health collaborative care management

This was a retrospective study among patients who receive their primary care in the Family Medicine and Primary Care Internal Medicine clinics within a large academic medical center in the Midwestern US, which provides primary care to over 140,000 patients at multiple sites. Approximately 2.1 % are patients with LEP, who require the use of a professional medical interpreter for clinical encounters. In 2008, these clinics implemented the Depression Improvement Across Minnesota, Offering A New Direction (DIAMOND) program, a statewide initiative that uses CCM for depression management [30]. This particular model has been well described [31], and includes weekly oversight by a psychiatrist, with medication or therapeutic changes managed by the primary care provider, and coordinated by a trained nurse care manager who interacts with the patient through face-to-face and telephone visits. Patients who meet DIAMOND criteria are referred to the program by their primary care providers, either by an in-person hand-off between the provider and nurse care manager within the context of a clinic visit, or through a phone referral when the nurse care manager is not immediately available. Patients then receive a comprehensive description of the program from the nurse care manager, after which they choose whether to enroll in the program or not. For patients with LEP, trained medical interpreters are used for all face-to-face and telephone interaction. This study (ref number: 13-009175) was approved by the Mayo Clinic Institutional Review Board (IRB).

### Study population

The DIAMOND patient registry was used for this study. Adults age ≥18 years, who were empanelled to a primary care provider in the practice, with a diagnosis of major depression (International Classification of Diseases-9 [ICD 9] code 296.2x, 296.3x) or dysthymia (ICD 9 code 300.4), and had a Patient Health Questionnaire-9 (PHQ-9) score ≥10 were eligible for enrollment. The PHQ-9 is a well-validated self-report of the frequency of symptoms for each of the 9 Diagnostic and Statistical Manual of Mental Disorders (4th edition) [32] criteria for depression that is easy to administer and interpret. It is used widely as a depression screening instrument in primary care settings [33].

Among patients in the DIAMOND registry, the cohort of patients who self-reported the need for interpreter services (IS) was identified by an administrative flag in the electronic medical record (EMR). IS need was used as a proxy for LEP, which has been done previously [34]. The final study cohort included all patients who met DIAMOND eligibility criteria between March 2008 and December 2013 (n = 7561), including both those who required IS (IS patients) and those who did not (non-IS patients). Only charts of patients who had given authorization for use of their medical records for research were included in the study.

### Data collection

The primary outcome measure was enrollment status into the DIAMOND program among the eligible patients. The following variables were obtained from the EMRs for each patient: age, gender, race, ethnicity, marital status, IS status, insurance type, education level, PHQ-9 score and medical complexity via the Charlson score [35].

### Data analysis

Descriptive analyses were used to compare the demographic characteristics by enrollment status in the DIAMOND program and by the IS status, using estimates of frequencies for categorical variables and medians and interquartile range for continuous variables. These were compared using a χ^2^ test for categorical variables and rank sum tests for continuous variables. The rate of enrollment into the DIAMOND program was calculated as the proportion of eligible patients who are enrolled into the DIAMOND program for each group. Logistic regression models were used to assess the association between the IS status and enrollment in the DIAMOND program, and results were presented as odds ratios (OR) and 95 % confidence intervals (CI). Multivariate logistic regression models were used to adjust for potential confounders, including age, gender, marital status, education, insurance, Charlson score and initial PHQ-9 score. Interactions with the IS status were assessed, but were non-significant and are therefore not reported. Low enrollment among IS patients into the DIAMOND program precluded comparative analysis of IS patients vs non-IS patients for DIAMOND outcomes (improvement in depressive symptoms; no change in depressive symptoms; worsening of depressive symptoms; loss to follow-up; and opt-out of program).

## Results

Of the 7561 patients who met criteria for enrollment into the DIAMOND program during the study interval, 3511 were enrolled. Compared to the non-enrolled DIAMOND-eligible patients, those who were enrolled were younger with higher baseline PHQ-9 scores, were more likely to have a college education, be privately insured, and had lower comorbidity scores.

Compared to the DIAMOND-eligible non-IS patients, DIAMOND-eligible IS patients (n = 77) were older, had a higher mean PHQ-9 score, were less likely to have a college education, less likely to have private insurance, and had higher comorbidity scores (Table 1).

**Table 1:** Patient characteristics by interpreter status

	Interpreter status		*P* value
	No (N = 7156)	Yes (N = 77)	
DIAMOND status (%)			<.01^a^
Enrolled	3376 (47.2)	14 (18.2)	
Not enrolled	3780(52.8)	63 (81.8)	
Age			.01^b^
Median	40.2	47.4	
Q1, Q3	28.6, 53.7	40.1, 56.6	
Gender (%)			.83^a^
Female	5008 (70.0)	61 (79.2)	
Male	2148 (30.0)	16 (20.8)	
Race (%)			<.01^a^
Black	160 (2.2)	11 (14.3)	
White	6611 (92.4)	14 (18.2)	
Other/unknown	290 (4.1)	33 (42.9)	
Asian	95 (1.3)	19 (24.7)	
Ethnicity (%)			<.01^a^
Hispanic or Latino	168 (2.3)	16 (20.8)	
Not Hispanic or Latino	6670 (93.2)	58 (75.3)	
Unknown	318 (4.4)	3 (3.9)	
Marital status (%)			.52^a^
Divorced/single/widowed	3481 (48.6)	24 (31.2)	
Married/partner	3613 (50.5)	53 (68.8)	
Other/unknown	62 (0.9)	0 (0.0)	
Insurance (%)			<.01^a^
Commercial	4972 (69.5)	20 (26.0)	
Medicaid	909 (12.7)	41 (53.3)	
Medicare	965 (13.5)	12 (15.6)	
Self-pay	250 (3.5)	3 (3.9)	
Other/unknown	60 (0.8)	1 (1.3)	
Education (%)			<.01^a^
≤High school	1946 (27.2)	52 (67.5)	
Some college≤	4847 (67.7)	14 (18.2)	
Unknown	363 (5.1)	11 (14.3)	
Charlson score (%)			<.01^a^
0	3337 (46.6)	30 (39.0)	
1	1947 (27.2)	19 (24.7)	
2	783 (11.0)	14 (18.2)	
>2	1087 (15.2)	14 (18.2)	
N/A	2	0	
Initial PHQ-9 score			.47^b^
Median	15.0	16.0	
Q1, Q3	12.0, 18.0	13.0, 19.0	

*DIAMOND* Depression Improvement Across Minnesota, Offering A New Direction, *PHQ-9* Patient Health Questionnaire-9

^a^Chi square

^b^Wilcoxon

Only 18.2 % of the eligible IS patients were enrolled into the DIAMOND program compared with the 47.2 % of the eligible non-IS patients (Table 1). Even after an adjustment for the differences in age, marital status, education, insurance status and Charlson score, IS patients were less likely to be enrolled in the DIAMOND program (adjusted OR [95 % confidence interval] = 0.43 [0.23, 0.81]) (Table 2).

**Table 2:** Association with Enrollment in the DIAMOND Program

	Unadjusted OR (95 % CI)	Adjusted OR (95 % CI)^a^
Interpreter status		
No	1.0	1.0
Yes	0.25 (0.14, 0.45)	0.43 (0.23, 0.81)
Gender		
Male	1.0	1.0
Female	1.33 (1.20, 1.47)	1.29 (1.15, 1.43)
Age		
18–28	1.0	1.0
29–40	1.25 (1.10, 1.41)	1.22 (1.05, 1.41)
41–53	1.02 (0.90, 1.16)	1.03 (0.89, 1.19)
54–97	0.89 (0.78, 1.01)	1.14 (0.96, 1.35)
Marital status		
Married/living together	1.0	1.0
Widowed/divorced/single	0.94 (0.86, 1.03)	1.02 (0.91, 1.13)
Other	1.35 (0.83, 2.20)	1.97 (1.13, 3.44)
Race		
White	1.0	1.0
Black	0.52 (0.38, 0.72)	0.66 (0.46, 0.94)
Asian	0.85 (0.59, 1.22)	0.82 (0.55, 1.24)
Other	0.61 (0.49, 0.76)	0.70 (0.54, 0.91)
Education		
Some college≤	1.0	1.0
≤High school	0.72 (0.65, 0.80)	0.87 (0.78, 0.98)
Insurance		
Commercial	1.0	1.0
Medicaid	0.65 (0.56, 0.75)	0.71 (0.61, 0.83)
Medicare	0.54 (0.47, 0.62)	0.65 (0.54, 0.78)
Self-pay	0.50 (0.37, 0.66)	0.49 (0.37, 0.67)
Charlson score		
0	1.0	1.0
1	0.96 (0.86, 1.08)	0.97 (0.86, 1.09)
2	0.80 (0.68, 0.93)	0.84 (0.71, 0.99)
>2	0.61 (0.53, 0.70)	0.77 (0.64, 0.92)
Initial PHQ-9	1.025 (1.013, 1.036)	1.035 (1.023, 1.047)

*CI* confidence interval, *DIAMOND* Depression Improvement Across Minnesota, Offering A New Direction, *OR* odds ratio, *PHQ-9* Patient Health Questionnaire-9

^a^Adjusted for interpreter status, gender, age, marital status, race, education, insurance, Charlson score, and initial PHQ-9

## Discussion

Within a large primary care practice that implemented CCM for depression, eligible IS patients were far less likely to enroll in the program than non-IS patients. This observation particularly warrants attention given that IS patients are more likely to seek and receive mental health services in primary care practices, rather than in specialty clinics [36, 37], and mental health CCMs embedded in primary care practices have been shown to be highly effective among patients with LEP [28]. These results suggest that mental health CCMs may run the risk of further widening existing disparities in mental health access and outcomes among these vulnerable populations.

Patients with LEP are heterogeneous in culture, ethnicity, race, and sociodemographic factors [38], and thus, the reasons underlying low utilization of mental health CCMs are likely multifaceted [37]. We could not attribute our findings on presence of traditional barriers to health care access and availability as most patients in this study had health insurance, were empanelled to a primary care practice, and were regular utilizers of outpatient clinics. Even after controlling for insurance type and education, IS patients were less likely to enroll. Other sociodemographic factors may be exerting a moderating effect.

Low socioeconomic position is highly correlated with mental health disorders [39–41], and is postulated to be both a cause and effect [42]. Identified barriers to utilization of mental health services among patients with low socioeconomic position and LEP include lack of transportation, inability to navigate complex health care systems, lack of trust on health care providers, and low health literacy [43–45]. Moreover, health care seeking behaviors among patients with LEP may be influenced by cultural norms, which may not align with mental health paradigms in the US [46, 47]. Stigma surrounding mental illness among some immigrant and refugee groups may also deter or delay seeking care [48, 49]. Organizational solutions to promote uptake of CCM services must therefore consider that LEP frequently coexists with low socioeconomic position, low health literacy, and culture-specific norms and values regarding mental health.

The utilization and efficacy of mental health CCMs relies on primary care providers’ awareness and acceptance of the program [24]. Furthermore, mental health disorders are more likely to manifest as somatic rather than emotive symptoms among immigrant and refugee patients. This leads to potential under-diagnosis by primary care providers and lost opportunities for referral to a CCM [49, 50]. Patient-provider relationships as well as the quality and experience of the interaction leading to the referral for CCM services, may also affect how much patients engage in the treatment for depression [51]. Together, these provider factors may contribute to lower referral of patients with LEP to CCM services.

While the CCM can be effective in improving mental health outcomes among patients with LEP, more research is needed to address the barriers to its optimal utilization among these patients. Incorporating elements that have been shown to improve health care access, utilization and outcomes among LEP patients, such as community engagement and incorporating language-congruent community health workers and navigators into CCM teams, may improve the utilization of these CCMs by patients with LEP [52–54]. Capacity building to enhance cultural competency and responsiveness within healthcare systems may help with patient engagement across the care continuum of immigrant and refugee patients and those with LEP [55].

The study is limited by its retrospective design and reliance on EMRs, though missing data were minimal. The use of IS need as a proxy for LEP is incomplete and represents only a subset of the true LEP patients. Furthermore, the fact that IS status is self-reported by patients at the time of registration may have led to misclassification. In addition, we were not able to verify the proportion of eligible patients who received IS during health care events, but institutional policy dictates that professional interpreters participate in every clinical encounter of patients with LEP. It was also not possible to control for or assess other factors that may have affected the enrollment of patients into the DIAMOND program, such as the quality of the patient-provider interaction and the mode of referral used. Though the small number of IS patients who enrolled in the CCM was an important and primary finding of the study, it precluded our ability to assess the efficacy of the CCM among patients with LEP. Finally, this study was conducted among patients seen at academically based primary care clinics in a single geographic region, with implications for generalizability.

## Conclusions

In summary, among patients empanelled to a large primary care practice, IS patients had significantly lower utilization of a mental health collaborative care model than patients who do not require IS. Understanding the reasons for this low rate of enrollment into the CCM is an important agenda for future research. Additional research is needed within primary care practices to implement socio-linguistically tailored interventions that improve the utilization of this model among IS patients.

## Abbreviations

CCM: collaborative care management
CI: confidence interval
DIAMOND: depression improvement across Minnesota, offering a new direction
EMR: electronic medical record
ICD-9: International Classification of Diseases-9
IS: interpreter services
LEP: limited english proficiency
PHQ-9: patient health questionnaire-9
OR: odds ratio
US: United States
